# Initial laboratory validation of temperature development models for *Necrodes littoralis* L. (Staphylinidae: Silphinae)

**DOI:** 10.1007/s00414-023-02969-4

**Published:** 2023-02-22

**Authors:** Joanna Gruszka, Szymon Matuszewski

**Affiliations:** 1 Laboratory of Criminalistics, Adam Mickiewicz University, Św. Marcin 90, 61-809 Poznań, Poland; 2 Center for Advanced Technologies, Adam Mickiewicz University, Uniwersytetu Poznańskiego 10, 61-614 Poznań, Poland; 3 Department of Animal Taxonomy and Ecology, Adam Mickiewicz University, Uniwersytetu Poznańskiego 6, 61-614 Poznań, Poland

**Keywords:** Forensic entomology, Insect age estimation, Post-mortem interval, PMI, Validation study

## Abstract

Development models of necrophagous insects are applied in forensic entomology for post-mortem interval estimation. Such estimates may be used as scientific evidence in legal investigations. For this reason, it is important that the models are valid and that the expert witness is aware of their limitations. *Necrodes littoralis* L. (Staphylinidae: Silphinae) is a necrophagous beetle species that frequently colonizes human cadavers. Temperature models of development for the Central European population of these beetles were recently published. In this article, we present results of the laboratory validation study for these models. Errors of beetle age estimation differed significantly between the models. Thermal summation models yielded the most accurate estimates, and the isomegalen diagram least accurate estimates. Errors of the beetle age estimation varied across beetle developmental stages and rearing temperatures. In general, most development models of *N. littoralis* were satisfactorily accurate in estimating beetle age under laboratory conditions; therefore, the study provides initial evidence to support their validity in forensic cases.

**Supplementary Information:**

The online version contains supplementary material available at 10.1007/s00414-023-02969-4.

## Introduction

Development models of necrophagous insects are used in legal investigations to yield an age of insect evidence sampled from human cadavers and eventually to estimate the minimum post-mortem interval (PMI_min_), which is the minimum time that elapsed from death to body disclosure [1]. Insect evidence can also be used to establish the circumstances of death in the case of endangered or protected wild animals [2]. Therefore, development models of forensically important species are used by forensic entomologists in their routine work. Since age estimates derived from these models are frequently used as scientific evidence in legal cases, the models should be valid [3]. Estimations based on unvalidated or flawed models may not only be challenged in court [4] but may also support unjust convictions in criminal trials [5]. Surprisingly, the validity of insect-based methods for the PMI estimation, and in particular the validity of development models for individual species, was rather poorly explored in validation studies [6].

PMI estimation protocols can be validated in several ways. The most common is the proof-of-assumptions study, which tests the basic assumptions of the protocol. The second type is the proof-of-concept study, where the protocol is tested in a simplified setup, usually under laboratory conditions. The least frequently used type is an experimental validation with human or non-human cadavers. It allows the protocol to be tested under experimental conditions, which can closely imitate conditions on a death scene. PMI estimation protocols can also be evaluated using forensic casework data [6]. Since development models of carrion insects are key elements of the protocols for the estimation of PMI based on insect development, validation types outlined above refer also to these models.

In recent years, efforts have been made to standardize the protocols used to sample and analyze insect evidence [7–10]. However, no attempts have been made to standardize methods used to create developmental models of necrophagous insects. Models are developed following various protocols, which in consequence can affect the accuracy and precision of age estimation using the models [4]. The creation of development models for a new species should be followed with their validation. The validation studies can objectively demonstrate the usefulness of a model for a specific purpose [5]. Knowing the limitations of the model, an expert may decide which method and model will be the most appropriate to the evidence at hand. In addition, validation studies frequently provide information on the precision and accuracy of age estimation using the model. Despite the awareness of the need to validate development models in forensic entomology [11–13], such studies are not common. Of the recently published models, only a few were validated (e.g., [14–18]).

*Necrodes littoralis* is a common necrophagous beetle with a Palearctic occurrence. Both larvae and adult insects are frequently found on human cadavers [19–24]. The first comprehensive development dataset for the Central European population of *N. littoralis* was recently published [25]. The dataset contains different types of temperature models that probably differ in the accuracy of insect age estimation. By analyzing the relative errors of age estimation using thermal summation models, isomorphen and isomegalen diagrams and growth curves, for beetles reared in the laboratory at five constant temperatures, we provide initial evidence to support the validity of these models. The current validation dataset exposes weaknesses and strengths of particular models when they are used to estimate the age of *N. littoralis*.

## Materials and methods

### Laboratory rearing and data collection

Data for the validation were collected from May 2021 to January 2022 using the same protocol as the one used for the modelling purposes [25]. However, we used a smaller number of rearing temperatures and smaller numbers of pupae and adult beetles. Moreover, the frequency of inspections (including measurements) was slightly lower.

To collect fresh eggs, adult beetles from our main colony were paired (two pairs per container) in plastic containers (18 cm × 11 cm × 14 cm) with soil, pork meat, and cotton wool with water. Containers were kept in temperature chambers (ST 1/1 BASIC or ST 1/1 + , POL-EKO, Poland) under five constant temperatures: 15, 18, 20, 22, and 26 °C. After oviposition, adult beetles were removed from the containers. Upon hatching, fifty fresh first instar larvae from each of the containers were transferred to new terrariums (the same size, soil, pork meat, and cotton wool with water). As a result, eight larval colonies (terrariums) were established per temperature. Once or twice a day (depending on larval stage and temperature), one larva from each of the eight containers was measured using a geometrical micrometer [26]. The larvae were measured in vivo and were returned to the container after the measurement. To ensure that a larva is fully extended, the measurement was taken in an Eppendorf tube while keeping it horizontally. Number of measurements were very small (12–20 measurements per colony during the whole study) to minimize the potential effects of in vivo measurements on the development. Post-feeding larvae were transferred to smaller containers (10 larvae per container) with soil for pupation. For this purpose, we looked for the moment when the larvae had finished feeding (in such case they are not present on the meat and do not walk on the soil surface, but bury themselves into the soil). Larvae placed in small, transparent containers usually form pupal chambers near the walls of the container, which allows for the monitoring of the further development and identification of the pupation and eclosion times.

Colonies were inspected for developmental landmarks (hatching, 1st and 2nd ecdysis, pupation, and eclosion) once, twice, or three times a day, depending on the temperature and the developmental stage. Since breeding was done in aggregations, the times to the first and the second ecdysis were determined for the colony. As there is variation in the timing of development within a colony, we recorded the beginning of the transition (when we observed the first post-ecdysis larva), the middle of the transition (when we observed that at least 50% of the larvae had moved to the next instar) and the end of the transition (when the last larva has moved to the next stage). These three timepoints were subsequently used in the analyses as the transition times for the colony. The times to the pupation and eclosion were monitored for individual beetles and therefore individual data were used in the analyses for these two landmarks.

### Validation

To validate thermal summation models, we compared the physiological age from the models (“model” *K*) with the true physiological age (“true” *K*) calculated using current data, according to the equation:

1 $$K=D\times \left(T-t\right)$$

where *D* is the duration of development, *T* is the rearing temperature, and *t* is the lower developmental threshold.

To assess the isomorphen diagram, we compared medians for developmental landmarks extracted from the diagram against the true transition times recorded in the present study. Evaluation of the isomegalen diagram and growth curves was performed similarly. The length of the larvae from this study was used to derive the times to reach these lengths in a given temperature according to the isomegalen diagram or a growth curve. Then, these times were compared with the true times recorded in this study.

For each model, we calculated relative errors of age estimation according to the formula:

2 $$\textrm{RELATIVE}\ \textrm{ERROR}=\frac{\mid \textrm{VALUE}\ \textrm{FROM}\ \textrm{THE}\ \textrm{MODEL}-\textrm{TRUE}\ \textrm{VALUE}\mid }{\textrm{TRUE}\ \textrm{VALUE}},$$

where VALUE FROM THE MODEL is *K* from the thermal summation model or median time for a given developmental landmark (from isomorphen diagram) or the time to reach a given larval length (from isomegalen diagram or growth curves) and TRUE VALUES are the true equivalents of these values as recorded in this study.

We used Kruskal-Wallis ANOVA to compare errors between the models, development stages and rearing temperatures (at *α* = 0.05, after Bonferroni correction 0.0056). Analyses were conducted in Statistica 13 (TIBCO Software Inc.).

## Results

**Fig. 1:**
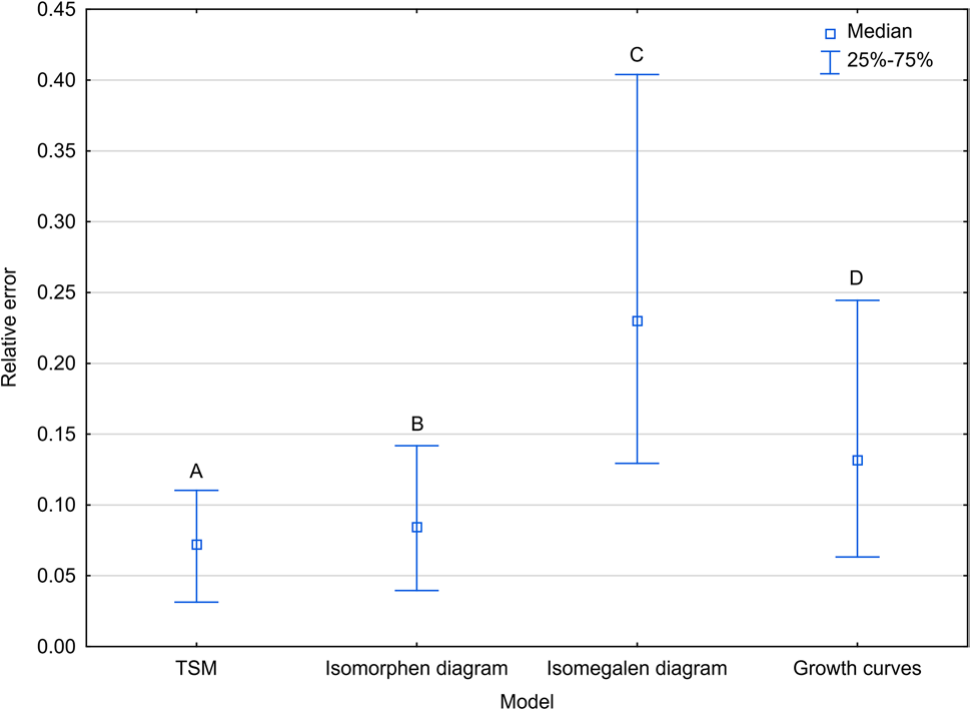
Relative error of age estimation in *Necrodes littoralis* using different developmental models. The graph shows the medians for all developmental stages. TSM – thermal summation models; Different letters denote significant differences in pairwise comparisons for Kruskal-Wallis ANOVA at *p* = 0.05

Errors of age estimation varied between the models, with the thermal summation model yielding the smallest errors and isomegalen diagram the largest errors (Kruskal–Wallis test: H(3) = 759.857, *p* < 0.001; Fig. 1; Supplementary Table 1).

**Fig. 2:**
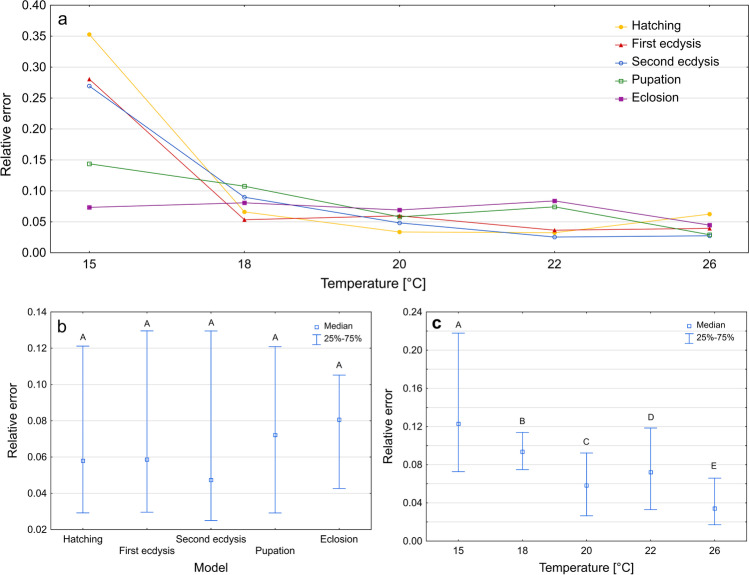
Relative error of age estimation in *Necrodes littoralis* using thermal summation models. **a** Medians of errors for developmental landmarks at each temperature. **b** Medians of errors for developmental landmarks only. **c** Medians of errors for temperatures only. Different letters denote significant differences in pairwise comparisons for Kruskal-Wallis ANOVA at *p* = 0.05

Thermal summation models produced differently accurate estimates of age depending on the rearing temperature (Kruskal–Wallis test: H(4) = 373.592, *p* < 0.001; Fig. 2c). The smallest errors were observed at the highest temperature and the largest errors at the lowest temperature (Fig. 2c, Supplementary Table 3). There were no significant differences in the relative error of age estimation between models for different developmental landmarks (Kruskal–Wallis test: H(4) = 1.332, *p* = 0.856; Fig. 2b).

The errors of age estimation using thermal summation models were in majority of cases below 0.1, regardless of the developmental event (70% of estimations for hatching, 68% for the first ecdysis, 69% for the second ecdysis, 65% for pupation, and 71% for eclosion). In the case of the thermal summation model for the eclosion, 93% of estimations had errors below 0.15 (Supplementary Fig. 1).

**Fig. 3:**
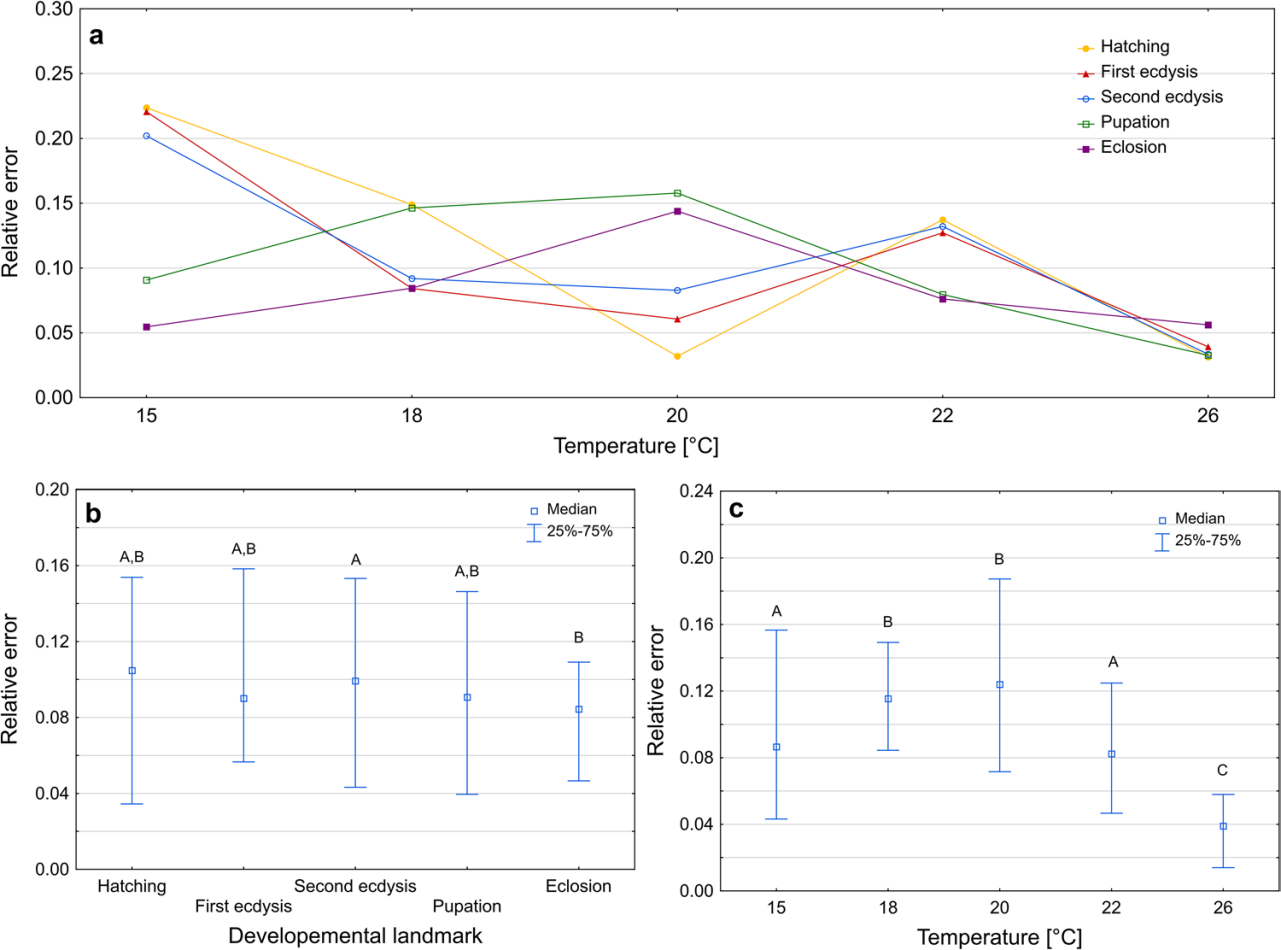
Relative error of age estimation in *Necrodes littoralis* using isomorphen diagram. **a** Medians of errors for developmental landmarks at each temperature. **b** Medians of errors for developmental landmarks only. **c** Medians of errors for temperatures only. Different letters denote significant differences in pairwise comparisons for Kruskal-Wallis ANOVA at *p* = 0.05

Isomorphen diagram gave different errors of age estimation depending on developmental landmark (Kruskal–Wallis test: H(4) = 14.871, *p* < 0.001, Fig. 3b) and rearing temperature (Kruskal–Wallis test: H(4) = 361,627, *p* < 0.001, Fig. 3c). Again, the smallest errors were recorded at the highest temperature (Fig. 3c). Most errors were below 0.1 (Supplementary Fig. 2).

**Fig. 4:**
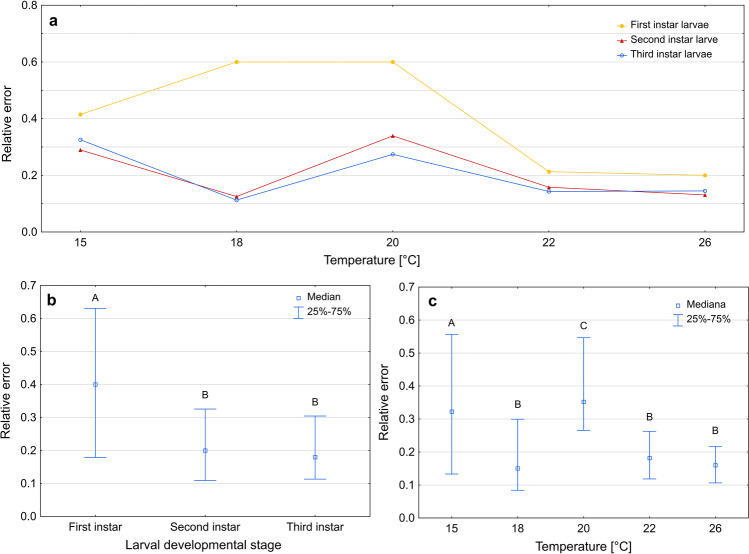
Relative error of age estimation in *Necrodes littoralis* using isomegalen diagram. **a** Medians of errors for larval developmental stages at each temperature. **b** Medians of errors for larval developmental stages only. **c** Medians of errors for temperatures only. Different letters denote significant differences in pairwise comparisons for Kruskal–Wallis ANOVA at *p* = 0.05

Estimating the age of larvae using an isomegalen diagram gave more accurate results for the second and the third instar larvae compared to the first instar larvae (Kruskal–Wallis test: H(2) = 73.945, *p* < 0.001; Fig. 4b; Supplementary Table 6). There were also differences in errors of age estimation between temperatures (Kruskal–Wallis test: H(4) = 111.206, *p* < 0.001; Fig. 4c, Supplementary Table 5).

**Fig. 5:**
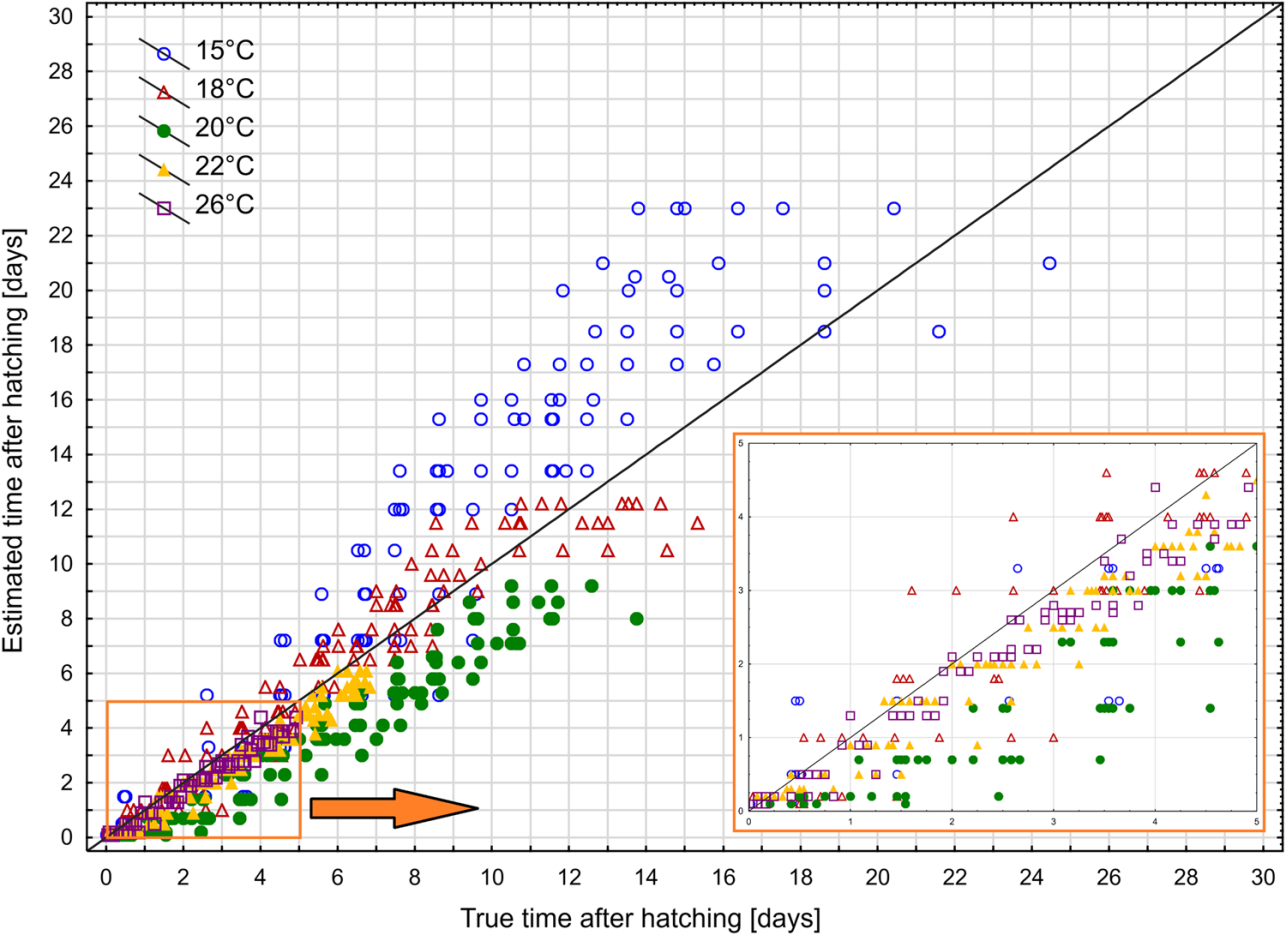
The estimated time after hatching (using isomegalen diagram) plotted against the true time after hatching. Solid line represents perfect estimates

In the case of the first instar larvae, 57% of relative errors of age estimation using the isomegalen diagram were below 0.5 and 92% were below 1. At least half of the age estimates for the second and the third instar larvae had errors lower than 0.1 (Supplementary Fig. 3). There were mostly overestimations at 15 °C, and underestimations at 20 °C, 22 °C, and 26 °C (Fig. 5).

**Fig. 6:**
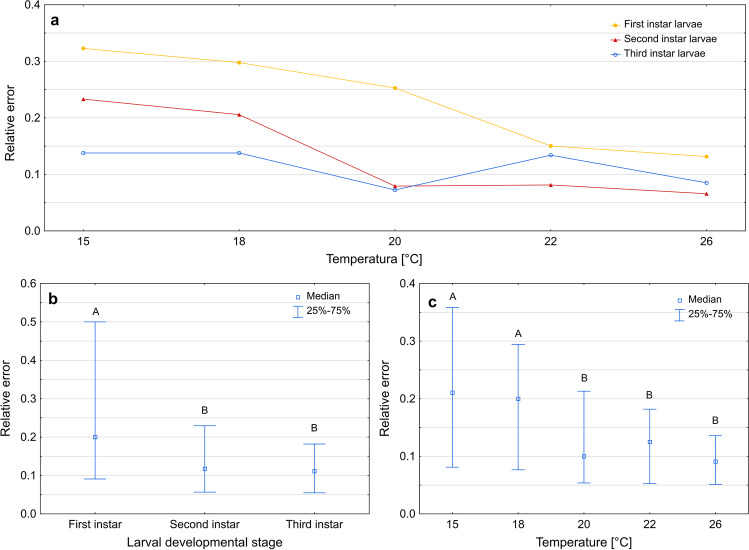
Relative error of age estimation in *Necrodes littoralis* using growth curves. **a** Medians of errors for larval developmental stages at each temperature. **b** Medians of errors for larval developmental stages only. **c** Medians of errors for temperatures only. Different letters denote significant differences in pairwise comparisons for Kruskal-Wallis ANOVA at *p* = 0.05

**Fig. 7:**
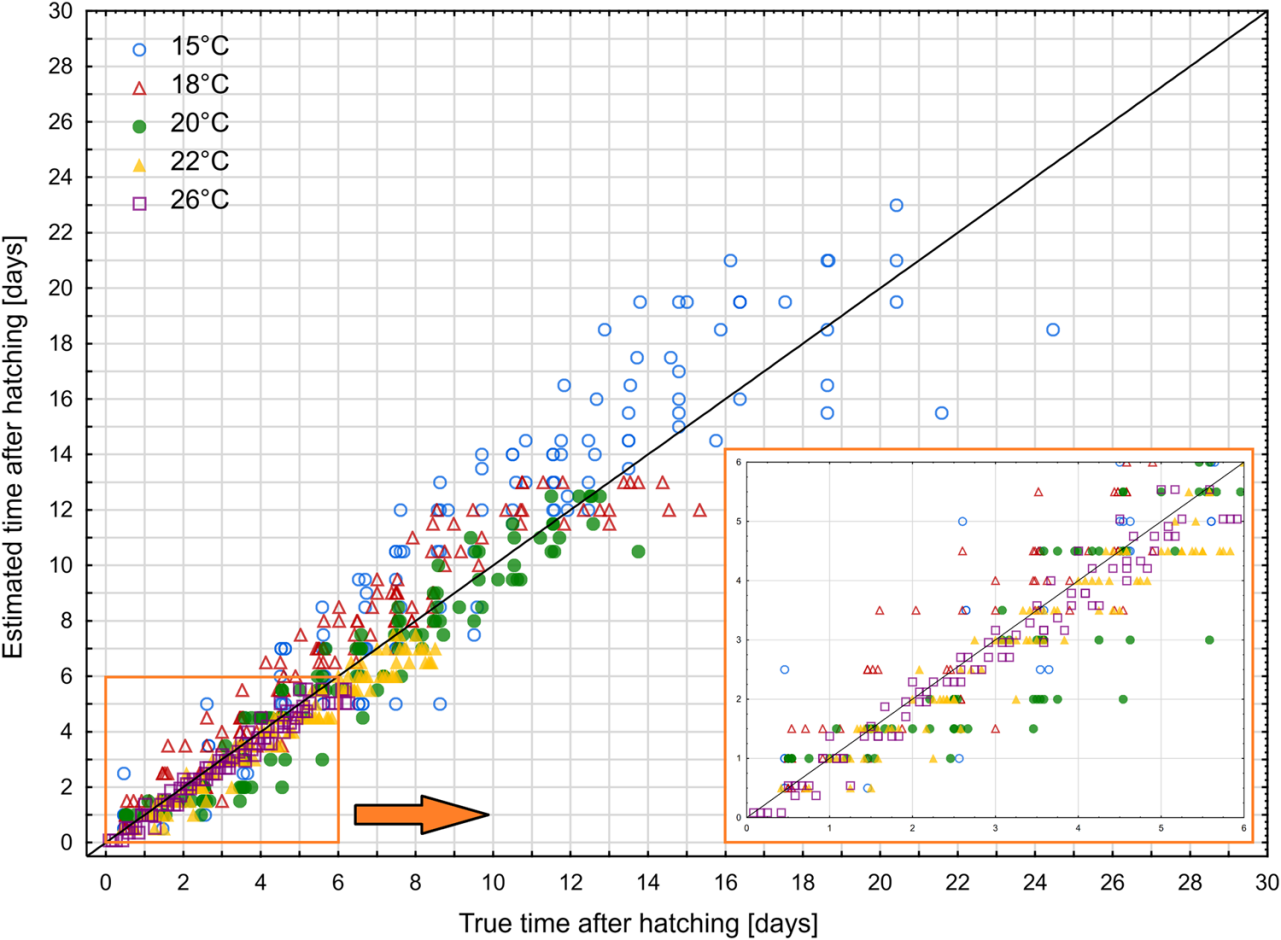
The estimated time after hatching (using growth curves) plotted against the true time after hatching. Solid line represents perfect estimates

Errors of age estimation using growth curves varied between developmental stages (Kruskal–Wallis test: H(2) = 43.437, *p* < 0.001) and temperatures (Kruskal–Wallis test: H(4) = 45,267, *p* < 0.001). The largest errors occurred in the case of the first instar larvae (Fig. 6b, Supplementary Table 8) and at 15 °C and 18 °C (Fig. 6c, Supplementary Table 9). At lower temperatures, the errors were mainly overestimations, at higher temperatures they were mostly underestimations (Fig. 7).

## Discussion

Our results indicate that the models based on the developmental landmarks, i.e., TSM and isomorphen diagram, allow for more accurate estimation of insect age than models based on the length of the larvae. The overall accuracy of the estimation is the result of a combination of precision and bias, and one of the sources of imprecision is the natural variation of the measured variable [11]. The variable relevant for the isomegalen diagrams and growth curves is the length of the larvae. It varies in larvae of the same age (i.e., larvae of the same length may be in different age) [11]. Moreover, the change in larval length may be more or less dynamic depending on the larval instar. High natural variation of larval length and poor correlation between length and larval age during the initial and final phases of the larval stage lower the final accuracy of the age estimation. In the case of the models based on developmental landmarks, this type of variation does not occur or has smaller effects. There is little natural variation in transitions between developmental stages and these transitions are closely related to insect age.

However, it should be noted that the estimation errors in the case of TSM and isomorphen diagrams relate to the age of insects exactly at the point of transition to the next developmental stage. In forensic casework, insect evidence are rarely found shortly after this transition; usually, they are collected sometime later. At low temperatures, it can be up to several days even in the case of the short developmental stages. For this reason, the estimation of insect age up to the developmental landmark with high accuracy does not mean that the estimate of the entire age of an insect will be similarly accurate. In such cases, developmental models based on larval length can be helpful, despite relatively large estimation errors; particularly, when no living insects were sampled [9].

For all the models, the temperature at which insects were reared had a more pronounced impact on the accuracy of age estimation than the developmental stage. Usually at higher temperatures, errors were smaller than at lower temperatures. The same pattern was observed in the case of *Creophilus maxillosus* [18]. These results suggest that the developmental models perform better at high temperatures. For TSM and isomorphen diagrams, this was likely because 15 °C was close to the temperatures at which the relationship between the rate of development and temperature is distinctly non-linear. On the other hand, 26 °C probably lies in the temperature range with a clearly linear relationship between the growth rate and the temperature [27]. In the case of growth curves and isomegalen diagrams, changes in larval length are simply more dynamic at high temperatures, and therefore, the accuracy of the estimation increases.

In the case of the isomegalen diagram and the growth curves, the age estimation errors of the first instar larvae were significantly larger than in the case of the second and the third instar larvae. First instar larvae grow at a lower rate than second and third instar larvae. Therefore, their length is less informative regarding larval age. Growth accelerates markedly in the second and particularly third larval stage, so in these stages, the length of a larva is a better predictor of its age than in the first larval stage. Moreover, the measurement errors, when using the Villet method [26] to measure larval length, are bigger for smaller larvae. Therefore, the first instar larvae are measured with less accuracy, which can lower the quality of a model and also the quality of validation data. In addition, the same absolute error (expressed in hours or days) when converted to a percentage value will constitute a larger relative error for the first instar larvae than for the second and third instar larvae [11].

Moreover, it should be remembered that development models are based on average values. Therefore, in both isomegalen diagrams and growth curves, there is a problem with the estimation of age for very small individuals. Either there will be a very large error in the estimation, or it will not be possible to estimate the age at all since extremely small larvae may be not included in the models. A similar limitation is with very large larvae which are significantly out of the largest average length (e.g., more than 23 mm for the isomegalen diagram and more than 25 mm for most growth curves). Although such long individuals are sometimes observed at peak growth, the average length for the population is smaller and the models (particularly isomegalen diagram) may not include such extreme lengths. Consequently, these models may be unsuitable for extremely small or large larvae. Therefore, models based on the length of *N. littoralis* larvae should be used with caution, especially when the insect evidence are the first instar larvae.

In addition, one of the problems in forensic entomology is that the studies leading to the construction of developmental models are laboratory studies at constant temperatures [28]. Under natural conditions, insects developing on cadavers experience fluctuations in temperature. The current validation dataset and the models that were initially validated here were also developed in the laboratory, and insects grew at constant temperatures. Therefore, further studies are necessary to give more insight into the validity and limitations of the models.

Another potential weakness of the development models for *N. littoralis* (and many other insects of forensic importance) is the use of the fresh meat as the food for the larvae. Recent studies indicated that *N. littoralis* reveal indirect forms of parental care. Adult beetles spread their exudates over carrion to form the feeding matrix that brings deferred thermal benefits for the larvae [29]. Moreover, adult *Necrodes* beetles were found to clear the carrion of the fly larvae to secure it for their offspring [30]. The latter behavior probably has no effect on the temporal patterns of larval development, but the preparation of the resource was found to shorten the development of the larvae [29]. At present, we are conducting a detailed study to quantify the differences in the timing of larval development between larvae fed with fresh meat and meat prepared by the adult beetles.

We are fully aware that the current study was only the first step in the validation process of the development models for N*. littoralis*. Ideally, validation using experimental data obtained from animal or human cadavers and validation based on case studies should be the next step to fully demonstrate the validity of the models*.* However, owing to the inherent difficulties, such studies were done only occasionally in forensic entomology [6]. Therefore, we encourage to validate the models at least in another study similar to the current one but performed in the different laboratory and using a different colony of *N. littoralis*.

While using thermal summation models for age estimation of *N. littoralis*, the relative error in most of the cases was below 10%. This suggests that the models allow for accurate estimation of beetle age and eventually PMI. However, *N. littoralis* is usually found on cadavers in an advanced stage of decomposition [19–21]. This means that insects of this species may colonize a cadaver many days after death. Their age, therefore, defines the minimum PMI. To get closer to the PMI, it is necessary in such cases to estimate the pre-appearance interval (PAI), which is the interval preceding appearance of an insect taxon on a cadaver [31]. The relative error of PAI estimation in *Necrodes littoralis* larvae using temperature methods is under 0.19 [32]. Accordingly, developmental models for *Necrodes littoralis* in combination with temperature methods for PAI should give a fairly accurate estimate of PMI.

It should also be remembered that the actual estimation of minimum PMI is based on several assumptions, and the errors inherent to these assumptions may affect the final accuracy of the minimum PMI [33]. The final result of the expert opinion will depend on the factors that directly affect the decomposition of a corpse and indirectly the development of insects, e.g., the temperature to which insects were exposed to [34], the type of habitat [35], body mass [36, 37], or drugs taken by the deceased [38].

Expert reports of forensic entomologists should contain a clear statement about variation in the estimation [27]. Hence, there is an urgent need to conduct validation studies and to publish case reports in this field, which may play an important role in learning more about the practical issues of forensic entomology [3]. It would also be useful to standardize methods for collecting laboratory development data and for deriving development models of forensically useful insects.

## Supplementary information

ESM 1

## Data Availability

The datasets generated and analyzed during the study are available from the corresponding author on a reasonable request.
