# Supplementary material for: Initial laboratory validation of temperature development models for Necrodes littoralis L. (Staphylinidae: Silphinae)
Source: Int J Legal Med. 2023 Feb 22;137(3):903–11. doi: 10.1007/s00414-023-02969-4 (PMC10085910; doi:10.1007/s00414-023-02969-4)
Supplement: Supplementary file 1 — ESM 1 [file 414_2023_2969_MOESM1_ESM.pdf]

Supplementary information for

Initial laboratory validation of temperature development models for *Necrodes littoralis* L. (Staphylinidae: Silphinae)

Joanna Gruszka[1,2,3*], Szymon Matuszewski[1,2]

[1] Laboratory of Criminalistics, Adam Mickiewicz University, Św. Marcin 90, 61-809 Poznań, Poland
[2] Center for Advanced Technologies, Adam Mickiewicz University, Uniwersytetu Poznańskiego 10, 61-614 Poznań, Poland
[3] Department of Animal Taxonomy and Ecology, Adam Mickiewicz University, Uniwersytetu Poznańskiego 6, 61-614 Poznań, Poland
[*]corresponding author: joanna.gruszka@amu.edu.pl

Supplementary tables

Supplementary Table 1. Relative error of age estimation in *Necrodes littoralis* using different developmental models. IQR - interquartile range, SE - standard error

Developmental model	Relative error of age estimation				N
	Median ± IQR	Mean ± SE	Minimum	Maximum	
Thermal summation model	0.072 ± 0.079	0.085 ± 0.002	0.000	0.910	1704
Isomorphen diagram	0.084 ± 0.102	0.098 ± 0.002	0.000	1.068	1703
Isomegalen diagram	0.230 ± 0.274	0.339 ± 0.017	0.000	3.800	601
Growth curves	0.131 ± 0.181	0.206 ± 0.011	0.000	4.454	622

Supplementary Table 2. Relative error of physiological age (K) estimation using thermal summation models for five developmental landmarks. IQR - interquartile range, SE - standard error

Model	Relative error of K estimation				N
	Median ± IQR	Mean ± SE	Minimum	Maximum	
Hatching	0.058 ± 0.092	0.113 ± 0.013	0.005	0.910	120
First ecdysis	0.059 ± 0.100	0.106 ± 0.011	0.001	0.499	120
Second ecdysis	0.047 ± 0.104	0.103 ± 0.011	0.000	0.521	120
Pupation	0.072 ± 0.092	0.083 ± 0.002	0.001	0.331	689
Eclosion	0.081 ± 0.062	0.076 ± 0.002	0.000	0.196	655

Supplementary Table 3. Relative error of physiological age (*K*) estimation using thermal summation models at five constant temperatures. IQR - interquartile range, SE - standard error

Temperature	Relative error of *K* estimation				N
	Median ± IQR	Mean ± SE	Minimum	Maximum	
15°C	0.123 ± 0.145	0.151 ± 0.006	0.000	0.521	329
18°C	0.094 ± 0.039	0.108 ± 0.005	0.010	0.910	293
20°C	0.058 ± 0.066	0.060 ± 0.002	0.001	0.199	332
22°C	0.072 ± 0.085	0.073 ± 0.002	0.001	0.203	372
26°C	0.034 ± 0.049	0.045 ± 0.002	0.000	0.137	378

Supplementary Table 4. Relative error of age estimation using isomorphen diagram depending on developmental landmark. IQR - interquartile range, SE - standard error

Model	Relative error of age estimation				N
	Median ± IQR	Mean ± SE	Minimum	Maximum	
Hatching	0.105 ± 0.119	0.120 ± 0.013	0.001	1.068	120
First ecdysis	0.090 ± 0.102	0.118 ± 0.009	0.000	0.550	120
Second ecdysis	0.099 ± 0.110	0.117 ± 0.008	0.000	0.440	120
Pupation	0.091 ± 0.107	0.099 ± 0.003	0.001	0.333	689
Eclosion	0.084 ± 0.062	0.086 ± 0.002	0.003	0.257	654

Supplementary Table 5. Relative error of age estimation using isomorphen diagram at five constant temperatures. IQR - interquartile range, SE - standard error

Temperature	Relative error of age estimation				N
	Median ± IQR	Mean ± SE	Minimum	Maximum	
15°C	0.086 ± 0.113	0.108 ± 0.005	0.001	0.440	329
18°C	0.115 ± 0.065	0.133 ± 0.006	0.016	1.068	293
20°C	0.124 ± 0.116	0.127 ± 0.004	0.000	0.333	333
22°C	0.081 ± 0.077	0.089 ± 0.002	0.003	0.194	371
26°C	0.039 ± 0.044	0.047 ± 0.002	0.000	0.150	377

Supplementary Table 6. Relative error of age (time from hatching) estimation using isomegalen diagram depending on developmental stage. IQR - interquartile range, SE - standard error

Developmental stage	Relative error of age estimation				N
	Median ± IQR	Mean ± SE	Minimum	Maximum	
First instar larvae	0.400 ± 0.459	0.540 ± 0.041	0.000	3.800	221
Second instar larvae	0.200 ± 0.217	0.229 ± 0.011	0.000	0.774	214
Third instar larvae	0.180 ± 0.191	0.214 ± 0.011	0.005	0.690	166

Supplementary Table 7. Relative error of age (time from hatching) estimation using isomegalen diagram at five constant temperatures. IQR - interquartile range, SE - standard error

Temperature	Relative error of age estimation				N
	Median ± IQR	Mean ± SE	Minimum	Maximum	
15°C	0.323 ± 0.423	0.482 ± 0.049	0.007	3.800	147
18°C	0.151 ± 0.215	0.288 ± 0.041	0.000	3.800	140
20°C	0.352 ± 0.281	0.416 ± 0.018	0.087	0.935	128
22°C	0.182 ± 0.144	0.215 ± 0.017	0.000	1.400	109
26°C	0.160 ± 0.111	0.209 ± 0.027	0.000	1.400	77

Supplementary Table 8. Relative error of age (time from hatching) estimation using growth curves depending on developmental stage. IQR - interquartile range, SE - standard error

Developmental stage	Relative error of age estimation				N
	Median ± IQR	Mean ± SE	Minimum	Maximum	
First instar larvae	0.200 ± 0.409	0.353 ± 0.032	0.000	4.454	189
Second instar larvae	0.118 ± 0.173	0.160 ± 0.009	0.000	0.578	214
Third instar larvae	0.111 ± 0.127	0.126 ± 0.006	0.000	0.442	219

Supplementary Table 9. Relative error of age (time from hatching) estimation using growth curves at five constant temperatures. IQR - interquartile range, SE - standard error

Temperature	Relative error of age estimation				N
	Median ± IQR	Mean ± SE	Minimum	Maximum	
15°C	0.211 ± 0.277	0.301 ± 0.036	0.000	4.454	147
18°C	0.200 ± 0.218	0.258 ± 0.024	0.000	1.769	134
20°C	0.100 ± 0.159	0.171 ± 0.017	0.000	1.000	127
22°C	0.125 ± 0.129	0.135 ± 0.010	0.000	0.667	123
26°C	0.091 ± 0.084	0.126 ± 0.014	0.000	0.800	91

Supplementary figures

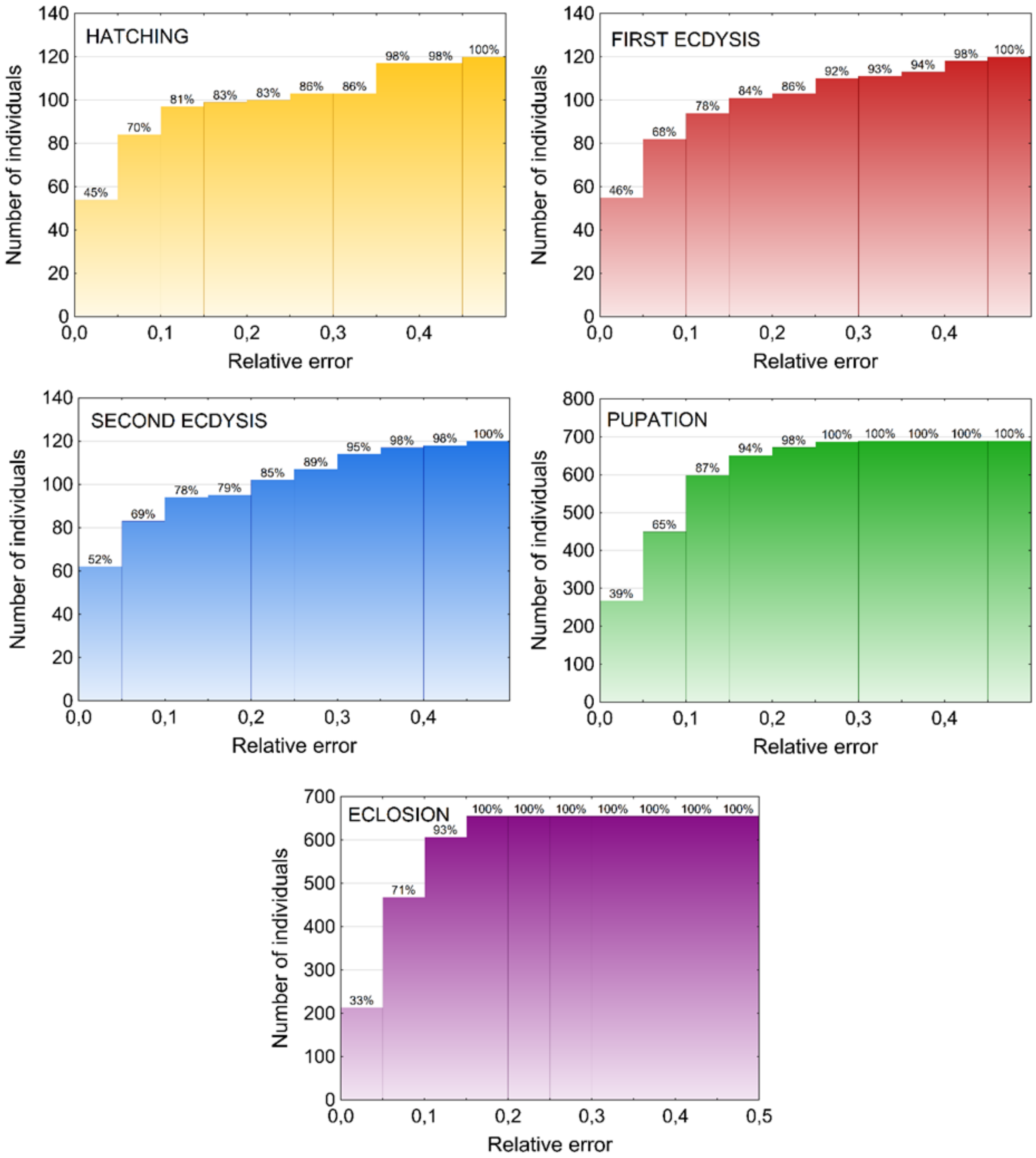

Supplementary Figure 1. Cumulative histograms for relative errors of physiological age (*K)* estimation, using thermal summation models for five developmental landmarks

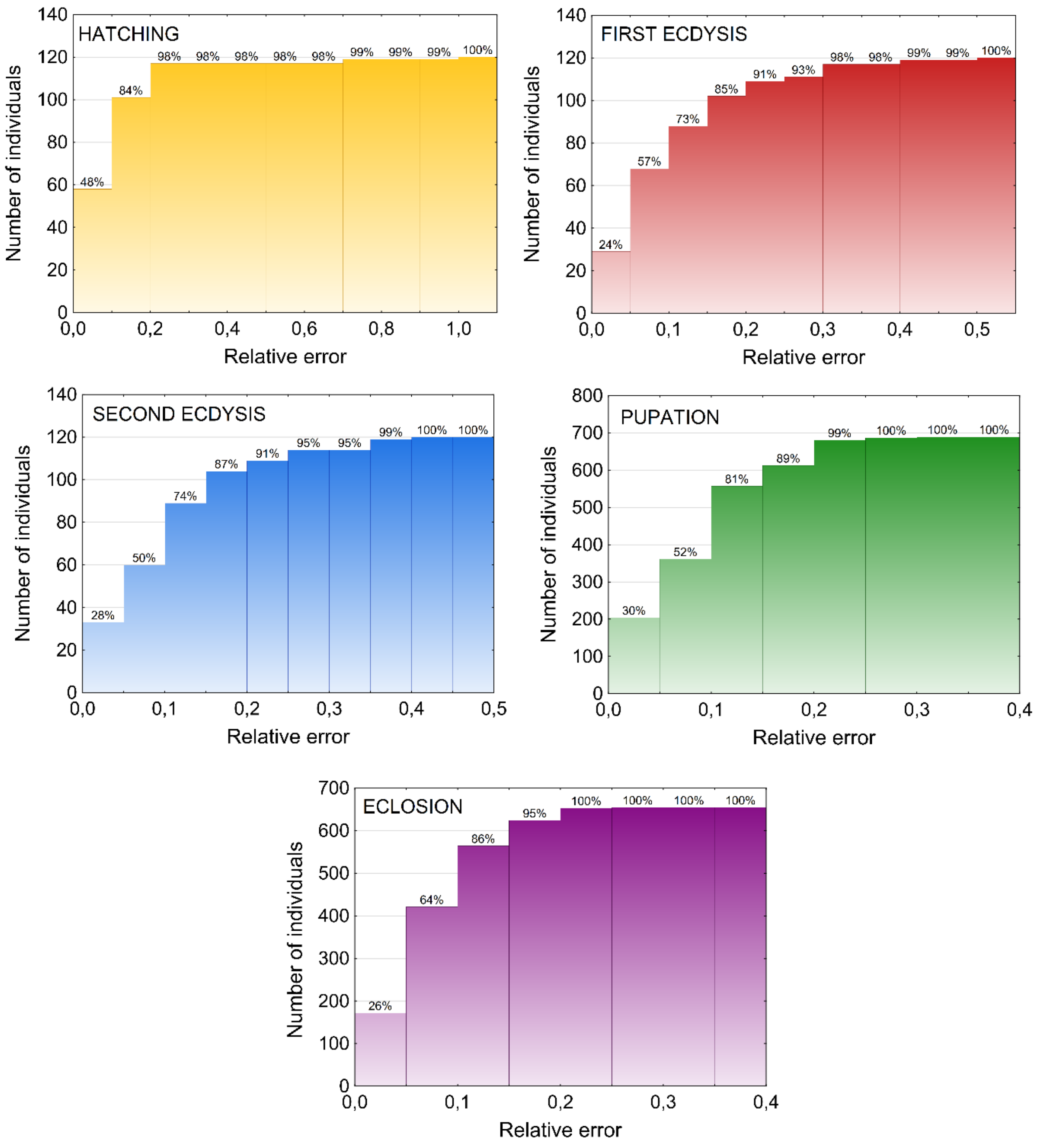

Supplementary Figure 2. Cumulative histograms for relative errors of age estimation, using isomorphen diagram.

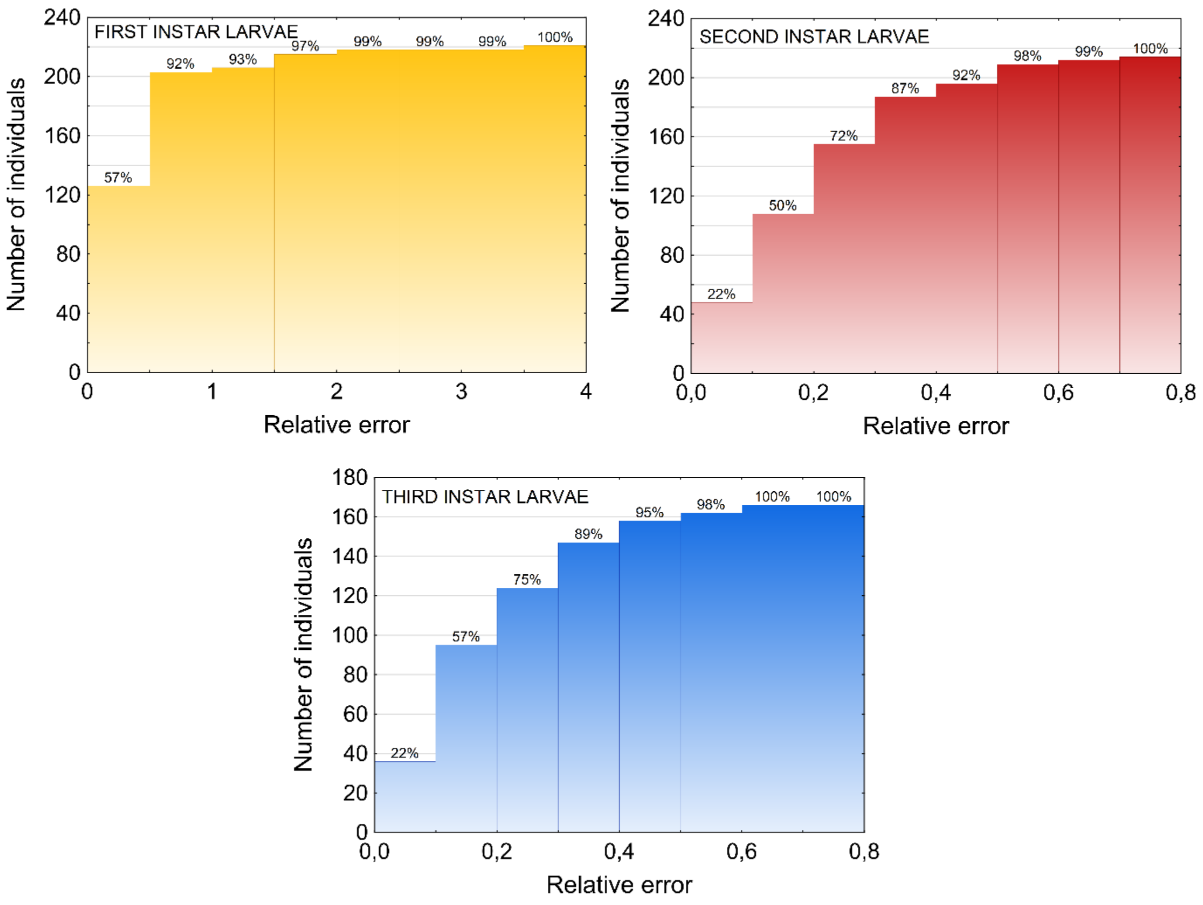

Supplementary Figure 3. Cumulative histogram for relative errors of age estimation (time from hatching) using isomegalen diagram, depending on the developmental stage.

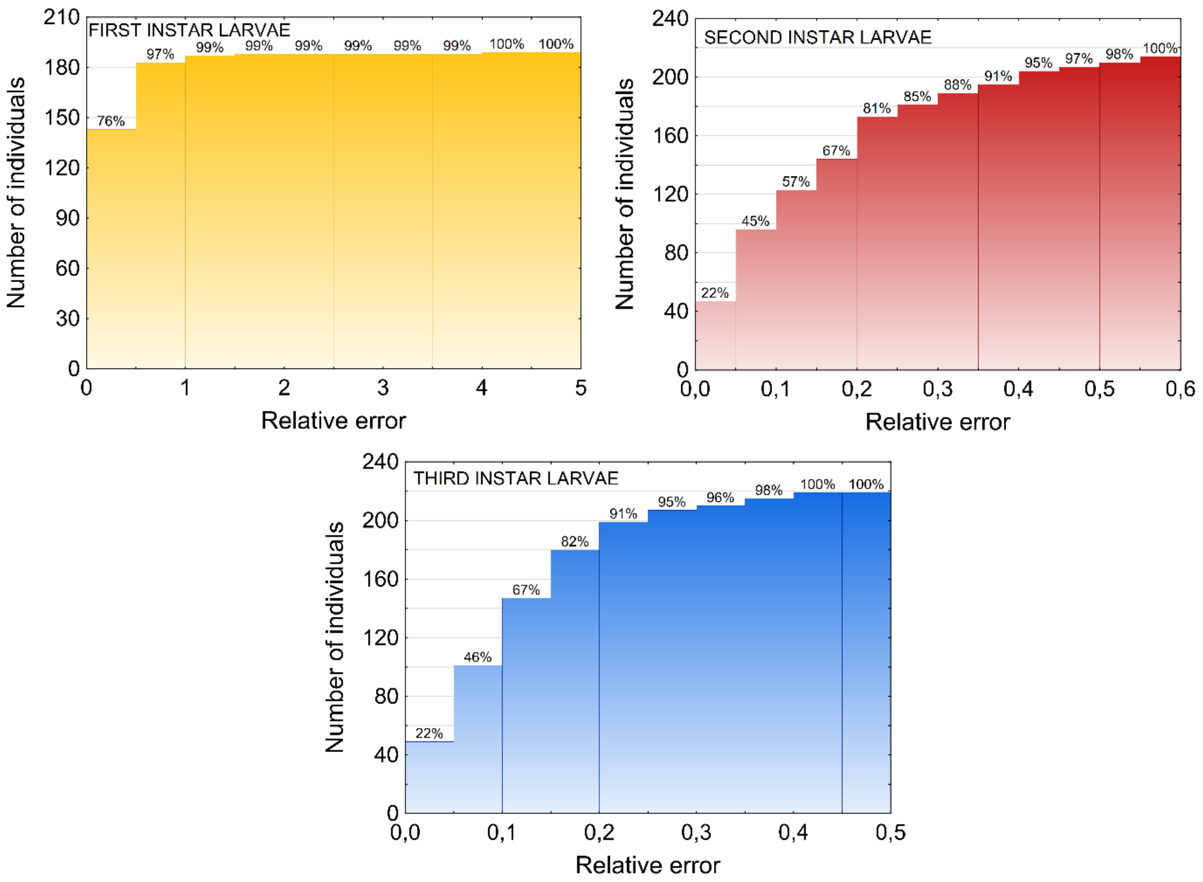

Supplementary Figure 4. Cumulative histogram for relative errors of age estimation (time from hatching) using growth curves, depending on the developmental stage.
